# PAK1 Mediates Bone Marrow Stromal Cell-Induced Drug Resistance in Acute Myeloid Leukemia via ERK1/2 Signaling Pathway

**DOI:** 10.3389/fcell.2021.686695

**Published:** 2021-07-08

**Authors:** Banban Li, Ruinan Jia, Wei Li, Ying Zhou, Dongmei Guo, Qingliang Teng, Shenghong Du, Mingying Li, Wěi Li, Tao Sun, Daoxin Ma, Min Ji, Chunyan Ji

**Affiliations:** ^1^Department of Hematology, Qilu Hospital, Cheeloo College of Medicine, Shandong University, Jinan, China; ^2^Department of Hematology, Taian City Central Hospital, Taian, China; ^3^Shandong Key Laboratory of Immunohematology, Qilu Hospital, Shandong University, Jinan, China

**Keywords:** p21-activated kinase 1, acute myeloid leukemia, bone marrow stromal cell, ERK1/2, drug resistance

## Abstract

**Background:**

Chemoresistance is emerging as a major barrier to successful treatment in acute myeloid leukemia (AML), and bone marrow stromal cells (BMSCs) protect leukemia cells from chemotherapy eventually leading to recurrence. This study was designed to investigate the role of p21-activated kinase 1 (PAK1) in AML progression and chemosensitivity, highlighting the mechanism of stroma-mediated chemoresistance.

**Methods:**

The GEPIA and TCGA datasets were used to analyze the relationship between PAK1 mRNA expression and various clinical parameters of AML patients. Cell proliferation and apoptosis were examined to evaluate the role of PAK1 on chemosensitivity in AML by silencing PAK1 with shRNA or small molecular inhibitor. Human BMSC (HS-5) was utilized to mimic the leukemia bone marrow microenvironment (BMM) *in vitro*, and co-culture model was established to investigate the role of PAK1 in BMSC-mediated drug resistance.

**Results:**

p21-activated kinase 1 high expression was shown to be associated with shorter overall survival in AML patients. The silence of PAK1 could repress cell proliferation, promote apoptosis, and enhance the sensitivity of AML cells to chemotherapeutic agents. More importantly, BMSCs induced PAK1 up-regulation in AML cells, subsequently activating the ERK1/2 signaling pathway. The effect of BMSC-mediated apoptotic-resistance could be partly reversed by knock down of PAK1.

**Conclusion:**

p21-activated kinase 1 is a potential prognostic predictor for AML patients. PAK1 may play a pivotal role in mediating BMM-induced drug resistance, representing a novel therapeutic target in AML.

## Introduction

Acute myeloid leukemia (AML) is a hematological malignancy characterized by proliferation of myeloid precursors with a reduced capacity to differentiate into more mature cellular elements in the bone marrow (BM), and is correlated with genetic and epigenetic alterations. Cytogenetic and molecular genetic abnormalities are responsible for determining the response to chemotherapy and survival outcome (Metzeler et al., 2016; Dohner et al., 2017). Although some improvement during the last decades has been made accompanied by current standard chemotherapy including anthracycline drugs and Cytarabine, the prognosis of AML is generally poor. The drug resistance and recurrence of leukemia are still more difficult issues for current treatment (Dombret and Gardin, 2016; Tallman et al., 2019). Therefore, further identification of drug-resistance relevant genes and signaling pathway provides insights into combinational targeted therapy.

The bone marrow microenvironment (BMM), which consists of soluble factors and supporting tissues, such as bone marrow stromal cells (BMSCs), extracellular matrix (ECM), provides a home for AML cells, and is responsible for disease relapse and treatment resistance (Shafat et al., 2017). BMM plays an important role in leukemia drug resistance by secreting various chemicals and contacting signals (Ayala et al., 2009). Previous studies have shown that many proteins were dramatically up-regulated in BMSC-conditioned AML cell lines, such as Galectin-3, cysteine-rich 61 and autophagy-related E1 ligase 7 (Hu et al., 2015; Long et al., 2015; Piya et al., 2016). However, the mechanisms of BMM-mediated drug resistance have been poorly elucidated.

The p21-activated kinase (PAK) family, belonging to serine/threonine kinases, is classified into group I (PAK1-3) and group II (PAK4-6) based on structural homology and regulatory function (Yao et al., 2020). Recently, the p21-activated kinase 1 (PAK1) has emerged as a potential therapeutic target in cancer, due to its key influence in a variety of oncogenic signaling pathways, including leukemia (Rane and Minden, 2014). Flis et al. (2019) demonstrated that the PAK1 inhibitor combined with BCR-ABL1 tyrosine kinase inhibitor displayed synergistic effect against chronic myeloid leukemia cells. Siekmann et al. (2018) also reported that combined inhibition of PAK1 and receptor tyrosine represented a valuable therapeutic strategy in childhood acute lymphoblastic leukemia (ALL). However, the role of PAK1 in BMSC-mediated drug resistance in AML has not been reported.

In the present study, we characterized PAK1 expression in AML patients using publicly available datasets to determine its association with their clinical and molecular characteristics and clinical outcome. High expression of total and phosphorylated (active) PAK1 in the majority of AML cell lines was observed. Additionally, the inhibition of PAK1 by genetic silencing could enhance the sensitivity of AML cells to chemotherapeutic agents. Moreover, PAK1 was dramatically up-regulated in BMSC-conditioned AML cell lines, accompanied by activation of ERK1/2 signaling. Collectively, our studies support that PAK1 mediates BMM-induced drug resistance via ERK1/2 signaling pathway in AML, which provides insights for the molecular mechanism of BMM-mediated treatment resistance and targeted or combined treatment.

## Materials and Methods

### TCGA RNA Sequencing and Clinical Data

The publicly available RNA sequencing data of 173 AML patients and complete clinical, molecular and survival data were downloaded from The Cancer Genome Atlas (TCGA) database. This database contains patients with previously untreated AML, all of them had been diagnosed, cytogenetics risk category according to the National Comprehensive Cancer Network (NCCN) guidelines. The RNA-seq and clinical data was analyzed using Gene Expression Profiling Interactive Analysis (GEPIA).

### Reagents and Antibodies

Cytarabine (Ara-C), Idarubicin (IDA), and dimethyl sulfoxide (DMSO) were purchased from Actavis (Nerviano, Italy) and Sigma (Merck, Germany), respectively. IPA-3 was obtained from Selleck (Houston, TX, United States). Antibodies of PAK1 and p-PAK1 was purchased from Abcam (MA, Cambridge, United States). GAPDH, ERK1/2, p-ERK1/2, β-actin and PARP, Cleaved-PARP antibodies were obtained from Cell Signaling Technology (Danvers, MA, United States).

### AML Cell Lines and Primary Samples

Five human AML cell lines THP1, Kasumi-1, U937, HL60, and KG1, a CML cell line K562, and a T-ALL cell line Jurkat (purchased from the Institute of Hematology and Blood Diseases Hospital, Chinese Academy of Medical Sciences, and Peking Union Medical College, Tianjin, China) were cultured in RPMI 1640 medium containing 10% heat-inactivated fetal calf serum with penicillin and streptomycin (all from Sigma) at 37°C with 5% CO_2_ in humidified incubator. The latest authentication of cell lines was conducted by Shanghai Zhong Qiao Xin Zhou Biotechnology Co., Ltd. (August to September, 2019). Bone marrow primary AML samples were collected from patients during routine diagnostic assessments in Qilu Hospital, Shandong University, Jinan, China. Control samples were obtained from healthy donors. Informed consent was obtained in accordance with the Declaration of Helsinki. Mononuclear cells from bone marrow aspirates were separated by Ficoll-Paque Plus (Pharmacia LKB Biotechnology) density gradient centrifugation.

### Lentiviral Transduction

The shRNAs against PAK1 in a lentiviral vector with green fluorescent protein, as well as the negative control were designed and synthesized by GeneChem (Shanghai, China). High-titer lentivirus was produced in 293T cells by transfection of the lentiviral expression vector and packaging vectors GV248 using a calcium phosphate cell transfection kit according to the manufacturer’s instructions (GeneChem, Shanghai, China). The lentivirus was harvested 48 h later, filtered, enriched using 40% polyethylene glycol, and then used to infect AML cells. After transfection for 72 h, the efficiency was estimated by evaluation of GFP expression by fluorescence microscopy and flow cytometry. The PAK1 specific shRNA sequence used in our study was 5′-CCAAGAAAGAGCTGATTATTA-3′. We used a flow sorter to sort the transfected cells expressing GFP fluorescence for subsequent experiments.

### Cell Viability Assay

Cell viability was determined by Cell Counting Kit-8 (CCK-8). CCK-8 was obtained from BestBio (Shanghai, China). Measurements were taken 24 h after drug exposure at the indicated concentrations. Absorbance was detected at 450/630 nm by a Microplate Reader (Bio-Rad).

### Cell Apoptosis Assay

Cell apoptosis was detected using an Annexin V-FITC/PI or Annexin V-FITC/7-AAD double stain apoptosis detection kit (BestBio, Shanghai, China) according to the manufacturer’s protocols.

### RNA Isolation and Quantitative RT-PCR Analysis

Total RNA was extracted from cells using the TRIzol reagent (Invitrogen, Carlsbad, CA, United States). Reverse transcription was performed with an M-MLV RTase cDNA Synthesis Kit (Takara, Japan). Real-time PCR was performed with the Roche Applied Science LightCycler 480 II Real-Time PCR System using the SYBR Green gene expression assay (Takara, Japan). The following primer sets were used (BioSune, China).

PAK1: 5′-CGCCCAGAGCACACAAAATC-3′ (forward)
5′-GTCCCGAGTTGGAGTGACAG-3′ (reverse)
GAPDH: 5′-GCACCGTCAAGGCTGAGAAC-3′ (forward)
5′-TGGTGAAGACGCCAGTGGA-3′ (reverse)

### Co-culture and Transwell Co-culture Experiments

For co-culture experiments, BMSCs (HS-5 cells) were seeded into 12 or 6-well plates at a density of 1 × 10^5^/ml overnight. Then, human AML cell lines or primary AML cells were seeded with HS-5 cells for 24 h. Cells were then treated with chemotherapy drugs or inhibitors for 24 h before performing Annexin V apoptosis assays. Co-cultured AML cells were separated from HS-5 monolayer by careful pipetting with ice-cold PBS (repeated twice). After collecting the leukemic cells, HS-5 cells monolayer was observed under microscopy (×100) to confirm that BMSC monolayer was not damaged and that <10 leukemic cells pervision field remained attached. As a control, AML cells were cultured alone and treated with chemotherapy drugs in parallel. For the transwell co-culture experiments, AML cell lines were plated inside the transwell microporous inserts (0.4 μm pore size) while underneath the inserts HS-5 cells were seeded into 12 or 6-well plates (ratio of AML cells: stromal cells, 1:1).

### Protein Extraction and Western Blot Analysis

Collected cells were lysed in lysis buffer and protease inhibitors. Fifteen to fifty micrograms of protein were separated by 10–12% sodium dodecyl sulfate-polyacrylamide gel electrophoresis (SDS-PAGE) and electrotransferred onto polyvinylidene fluoride membranes. The membranes were blocked in 5% bovine serum albumin (BSA) at room temperature for 2 h, incubated with primary antibodies overnight at 4°C, and then incubated with a secondary antibody at room temperature for 1 h. Immunoreactive bands were visualized using an infrared imaging system.

### Immunofluorescence

The cell lines which were washed with PBS were plated onto cover-slips and fixed with 4% paraformaldehyde solution for 10 min. Permeabilize cells by incubating with 0.1% Triton X-100 for 15 min. All slides were then washed with PBS and blocked with Goat serum at room temperature for 30 min. Cells were incubated with primary antibodies at 4°C overnight in a dark humidity chamber. Washed samples with PBS three times, and then incubated cells with secondary antibodies for 1 h at room temperature. In the dark, dye with DAPI for 10 min. Cells were examined immediately using Evos Fiber Illuminator (Invitrogen, United States) fluorescence microscope.

### Statistical Analysis

Statistical analysis were performed using SPSS V20.0. Data were presented as mean ± standard deviation (SD) from at least three independent experiments. Statistical differences between two groups were evaluated using Student’s *t*-test (paired or unpaired, as appropriate), and one-way ANOVA was used to determine the significant differences among multiple groups. Mann–Whitney *U* test was used for cases with unequal variances. Pearson’s Chi-square test was used to compare the clinicopathological characteristics between groups. Survival was presented with a Kaplan-Meier survival plot. Spearman correlation test was used to test the correlation between gene expressions. Differences were considered statistically significant at *P* < 0.05.

## Results

### PAK1 Is Highly Expressed in AML Patients and Cell Lines

**FIGURE 1:**
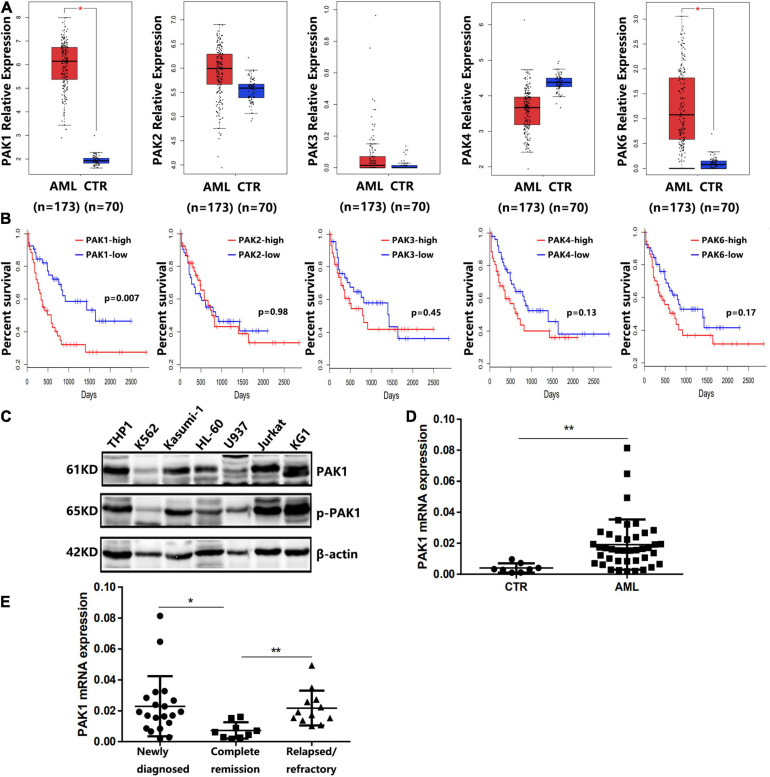
Expression of PAK family members in AML patients and cell lines. **(A)** The expression levels of PAK family members in AML patients (*n* = 173) and normal controls (CTR, *n* = 70) from the TCGA database. **(B)** Overall survival of AML patients from TCGA database. AML patients were divided into low and high groups based on the median expression value of PAK1-6. **(C)** The expression of PAK1 and phosphorylated PAK1 at Ser144 (P-PAK1) in seven leukemia cell lines by western blot analysis. THP1, Kasumi-1, U937, HL60, and KG1 are AML cell lines, K562 is a CML cell line, and Jurkat is a T-ALL cell line. β-actin was used as an internal loading control. **(D)** Quantitative mRNA expression of PAK1 by RT-qPCR in AML patient samples (*n* = 32) and controls (CTR, *n* = 8). **(E)** Quantitative mRNA expression of PAK1 in patients with newly diagnosed AML (*n* = 20), patients with relapsed/refractory AML (*n* = 12) and patients with complete remission (*n* = 9). *P < 0.05, **P < 0.01.

The RNA-seq dataset of 173 adult newly diagnosed AML patients and corresponding clinical profiles were obtained from TCGA database. Detailed clinical and molecular characteristics of them are shown in Table 1. Using GEPIA, we compared the mRNA expression levels of PAK family members between AML patients (*n* = 173) and normal controls (*n* = 70). The results showed that the expression levels of PAK1 and PAK6 were significantly higher in AML patients than in normal controls (*P* < 0.05, Figure 1A). However, the expression levels of other PAK family members, including PAK2, PAK3, and PAK4, were not statistically different between AML patients and normal controls (Figure 1A). We further divided AML patients into two groups based on the median expression value of PAK1-6, and the survival of AML patients in different groups were analyzed. The results showed that only PAK1 high expression was associated with poor overall survival (OS) in AML patients (*P* = 0.007, Figure 1B). Moreover, we detected the expression of PAK1 and phosphorylated PAK1 at Ser144 (p-PAK1) in seven leukemia cell lines (THP1, Kasumi-1, U937, HL60, and KG1 are AML cell lines, K562 is a CML cell line, and Jurkat is a T-ALL cell line) by western blot analysis. As illustrated in Figure 1C, PAK1 was highly expressed and phosphorylated in the majority of AML cell lines. PAK1 mRNA levels were detected in the bone marrow samples of AML patients (*n* = 32) and control samples (CTR, *n* = 8). PAK1 expression was significantly increased in AML patients compared to control (*P* < 0.01, Figure 1D). PAK1 expression was higher in patients with newly diagnosed AML (*n* = 20) and relapsed/refractory AML (*n* = 12) than in patients with complete remission (*n* = 9, *P* < 0.05, Figure 1E).

**TABLE 1:** Association of PAK1 expression with clinical characteristics of AML patients.

**Characteristics**	**Overall Cohort (*n* = 173)**		***P*-Value**
	**PAK_High**	**PAK_Low**	
	***n* = 99**	***n* = 74**	
Gender (male/female)	58/41	35/39	0.1423
Age: median (range)	61 (18–88)	53 (21–82)	0.0070
Peripheral blast,% median (range)	70 (0–98)	72 (0–100)	0.6416
Bone marrow blast,% median (range)	18 (0–96)	48 (0–98)	0.0020
WBC count (10^9^/L), median (range)	19 (1–224)	14 (1–297)	0.8964
Hemoglobin (g/dL), median (range)	9 (6–13)	10 (6–14)	0.0500
Platelet count (10^9^/L), median (range)	57 (11–351)	37 (8–232)	0.0082
**FAB classification (n)**			
M0	10	6	0.6544
M1	19	23	0.0712
M2	20	19	0.3940
M3	0	16	<0.0001
M4	32	3	<0.0010
M5	16	2	0.0041
M6	1	1	0.8354
M7	1	2	0.3988
NA	0	2	0.1000
Cytogenetic risk group (n)			<0.0001
Favorable	6	26	<0.0001
Intermediate/normal	67	36	0.0116
Poor	26	10	0.0410
NA	0	2	0.1000
**Molecular abnormality**			
FLT3 mutation			0.0099
Positive	21	29	
Negative	74	39	
NA	4	6	
**IDH1 mutation**			0.8678
Positive	17	12	
Negative	80	57	
NA	2	5	
**RAS mutation**			0.9172
Positive	5	4	
Negative	94	67	
NA	0	3	
**NPMc mutation**			0.7108
Positive	23	19	
Negative	76	52	
NA	0	3	
**Cytogenetic abnormality (n)**			
Normal	54	34	0.2630
Complex	17	5	0.0419
Trisomy 8	5	4	0.9172
del (7q)/7q−	5	2	0.4381
Inv. (16)	3	4	0.4328
*t* (8;21)	1	6	0.0191
*t* (15;17)	0	11	<0.0010
Others	2	3	0.4295
NA	12	5	0.2409

### PAK1 High Expression Is Associated With Poor Prognosis of AML Patients

**FIGURE 2:**
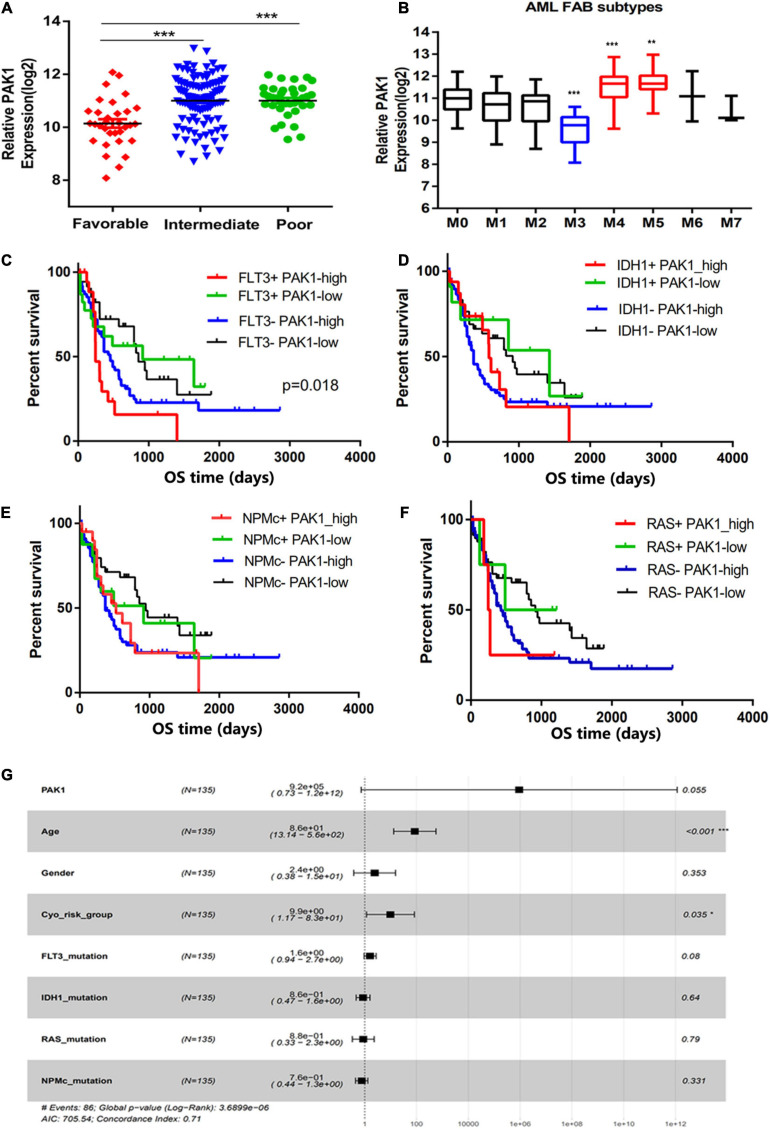
p21-activated kinase 1 (PAK1) high expression is associated with poor prognosis of AML patients. **(A)** The PAK1 expression of AML patients in different cytogenetic risk status from TCGA database. **(B)** The PAK1 relative expression in distinct AML FAB subtypes according to cytomorphological classification. **(C–F)** Significance of PAK1 expression in the prognosis of AML patients with genomic mutations, such as FLT3, IDH1, NPMc, and RAS mutations. AML patients were divided into four groups according to genomic mutation status and PAK1 expression level. **(G)** Forest map of COX multi-factor analysis. *P < 0.05, **P < 0.01, ***P < 0.001.

To explore the significance of PAK1 in AML, we first divided AML patients into low and high groups based on PAK1 median expression value. Characteristics of 99 patients with high PAK1 expression and 74 patients with low PAK1 expression were analyzed (Table 1). High expression of PAK1 was shown to be closely associated with older age and higher cytogenetic risk. We found that patients in the favorable group had significantly lower PAK1 expression compared with other groups (*P* < 0.001, Figure 2A). In addition, patients of M4 and M5 subtypes had relatively higher PAK1 expression, but patients of M3 subtype, which was the best type of AML, had significantly lower PAK1 expression (*P* < 0.001, Figure 2B). The significance of PAK1 expression implicated in the prognosis of AML patients with genomic mutations and cytogenetic abnormalities was also analyzed. As shown in Table 1, FLT3 mutation was more frequent in patients with low PAK1 expression. However, patients with high PAK1 expression tended to have more complex karyotype (*P* = 0.0419), but less *t* (8;21) and *t* (15;17) cytogenetic abnormalities (*P* = 0.0191, *P* < 0.001). Furthermore, high PAK1 expression was shown to be associated with poorer prognosis in AML patients without FLT3, IDH1, NPMc, or RAS mutation (*P* < 0.05, Figures 2C–F). For FLT3 mutation status, there was statistical difference among patients with high or low PAK1 expression (*P* = 0.018). Specifically, patients with FLT3 mutation and high PAK1 expression had the shortest survival time, though this was not statistically different from patients with no FLT3 mutation and high PAK1 expression. COX multi-factor analysis showed that, similar to the age and cytogenetic risk status, PAK1 might be an independent prognostic factor for AML (*P* = 0.055, Figure 2G).

### PAK1 Suppression Inhibits Cell Growth and Promotes Apoptosis of AML Cells

**FIGURE 3:**
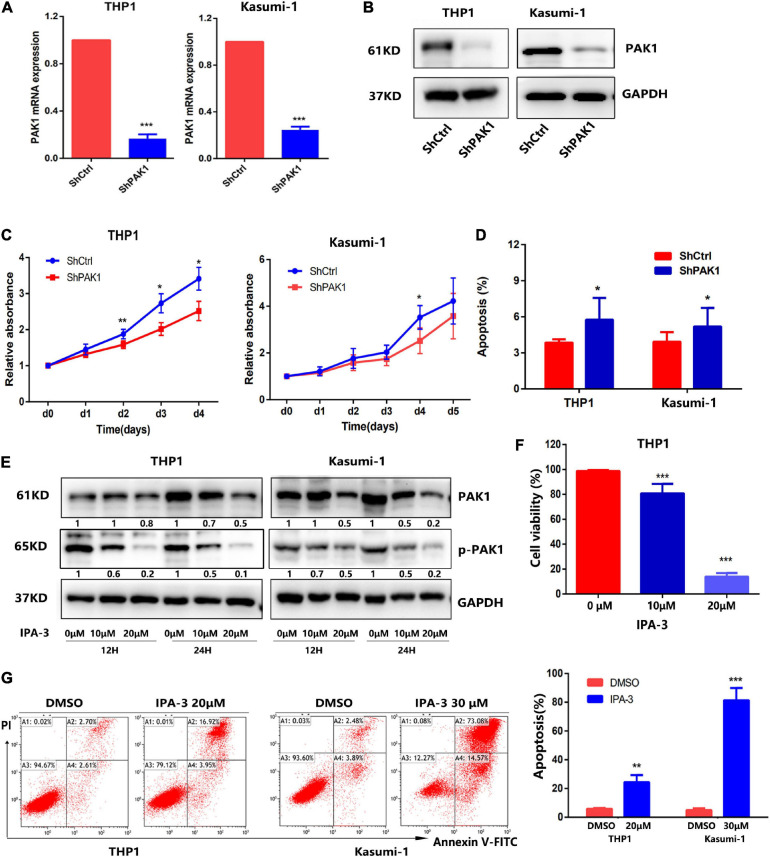
p21-activated kinase 1 (PAK1) suppression inhibits cell growth and promotes apoptosis of AML cells. **(A,B)** THP1 and Kasumi-1 cells were infected with lentiviral particles with specific PAK1 shRNAs (ShPAK1) or scrambled control (ShCtrl) for 48 h. PAK1 mRNA was detected by RT-qPCR **(A)** and the protein of PAK1 was detected by western blot **(B)**. **(C)** After PAK1 suppression, the proliferation of THP1 and Kasumi-1 cells (shPAK1 or shCtrl) was assessed by CCK-8 assays. **(D)** After PAK1 suppression, the apoptosis of THP1 and Kasumi-1 cells (shPAK1 or shCtrl) was detected by flow cytometry. **(E)** THP1 and Kasumi-1 cells were treated with IPA-3 (0, 10, and 20 μM) for 12 and 24 h, respectively. The expression of PAK1 and p-PAK1 was detected by western blot. **(F)** THP1 cells were treated with 10 and 20 μM IPA-3 for 24 h and the cell viability was detected by CCK-8 assay. **(G)** THP1 and Kasumi-1 cells were treated with 20 and 30 μM IPA-3 for 24 h, respectively. Cell apoptosis was evaluated by flow cytometry. All the experiments were repeated for at least three times. *P < 0.05, **P < 0.01, ***P < 0.001.

To explore the functional significance of PAK1 in AML, we firstly downregulated PAK1 expression in THP1 and Kasumi-1 cells by lentivirus with shRNAs that targeted PAK1 (ShPAK1). Transfection efficiency was more than 95% evaluated by fluorescence microscope and flow fluorescence analysis (Supplementary Figure 1). RT-qPCR and western blot showed that PAK1 mRNA and protein levels were significantly downregulated in THP1 and Kasumi-1 cells (Figures 3A,B). We then verified the effects of PAK1 suppression on cell proliferation and apoptosis. The results showed that after PAK1 suppression by shRNA lentivirus, cell growth was inhibited in both THP1 and Kasumi-1 cells, and cell apoptosis was increased (Figures 3C,D).

To better understand the role of PAK1 in AML, a selective non-ATP competitive PAK1 small molecule inhibitor IPA-3 (Rane and Minden, 2014; Yao et al., 2020) was used to suppress PAK1 expression. THP1 and Kasumi-1 cells were treated with IPA-3 (0, 10, and 20 μM) for 12 and 24 h, respectively. Western blot showed that IPA-3 significantly reduced the expression of p-PAK1 and PAK1 (Figure 3E). Functional assays revealed that PAK1 silencing by IPA-3 could remarkably suppress cell viability of THP1 cells and increase the apoptosis of THP1 and Kasumi-1 cells (Figures 3F,G). These results suggested that the silence of PAK1 could inhibit cell proliferation and promote apoptosis of AML cells.

### PAK1 Suppression Enhances Chemotherapy Drugs Induced Apoptosis of AML Cells

**FIGURE 4:**
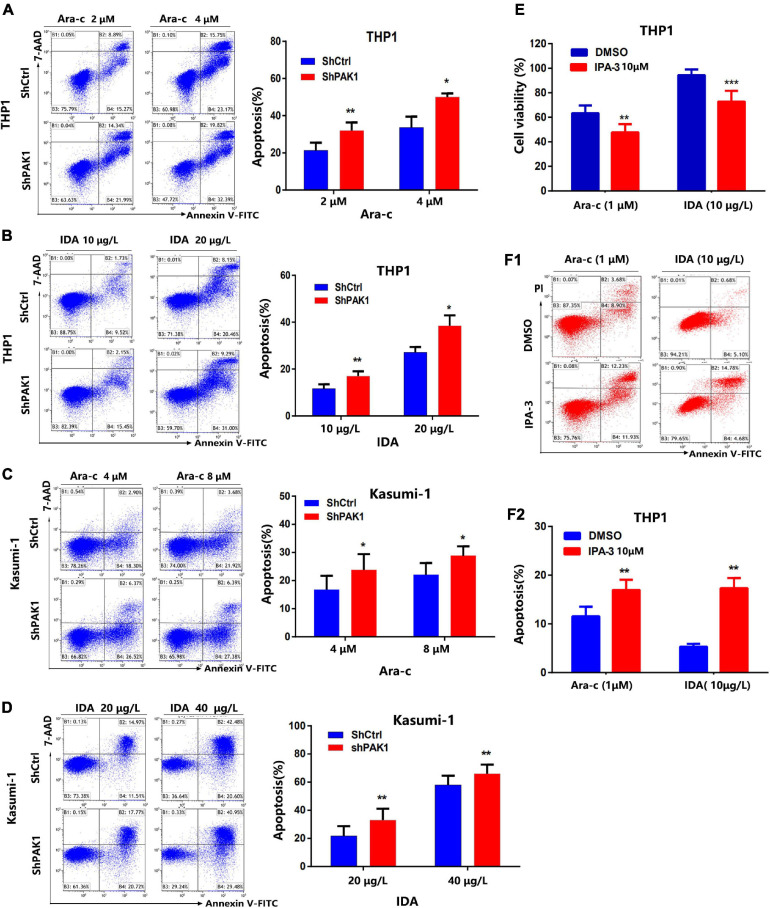
p21-activated kinase 1 (PAK1) suppression enhances chemotherapy drugs induced apoptosis of AML cells. **(A,B)** After PAK1 suppression, THP1 cells (shPAK1 or shCtrl) were treated with different concentrations of Ara-C (0, 2, and 4 μM) or IDA (0, 10, and 20 μg/L) for 24 h, and flow cytometry was performed to determine the apoptosis of AML cells induced by chemotherapy drugs. **(C,D)** After PAK1 suppression, Kasumi-1 cells (shPAK1 or shCtrl) were treated with different concentrations of Ara-C (0, 4, and 8 μM) or IDA (0, 20, and 40 μg/L) for 24 h, and flow cytometry was performed to determine the apoptosis of AML cells induced by chemotherapy drugs. **(E)** THP1 cells were treated with 10 μM IPA-3 and Ara-C (1 μM) or IDA (10 μg/L) for 24 h. The cell viability was detected by CCK-8 assay. **(F1,F2)** THP1 cells were treated with IPA-3 (10 μM) and Ara-C (1 μM) or IDA (10 μg/L) for 24 h. Flow cytometry was performed to determine the apoptosis of THP1 cells induced by chemotherapy drugs. All the experiments were repeated for three times. *P < 0.05, **P < 0.01, ***P < 0.001.

It is well known that resistance to chemotherapy is the main reason for the relapse and refractory of leukemia, which resulted in fast progression of disease. Based on the fact that AML patients with PAK1 overexpression had shorter overall survival, we further explored the effect of PAK1 on drug-induced apoptosis. After PAK1 suppression by lentivirus with shRNA, THP1, and Kasumi-1 cells were treated with different concentrations of Ara-C (THP1: 0, 2, and 4 μM; Kasumi-1: 0, 4, and 8 μM) or IDA (THP1: 0, 10, and 20 μg/L; Kasumi-1: 0, 20, and 40 μg/L) for 24 h, and flow cytometry was performed to determine the apoptosis of AML cells induced by chemotherapy drugs. The results showed that knockdown of PAK1 could markedly increase Ara-C and IDA induced apoptosis of AML cells in a dose-dependent manner (Figures 4A–D). Our data indicated that downregulation of PAK1 could significantly enhance the sensitivity of AML cells to chemotherapy drugs.

THP1 cells were treated with IPA-3 (10 μM) and Ara-C (1 μM) or IDA (10 μg/L) for 24 h. The cell viability was detected by CCK-8 assay (Figure 4E). Flow cytometry was performed to determine the apoptosis of THP1 cells induced by chemotherapy drugs with or without IPA-3 (Figure 4F1-2). Our data suggest that the PAK1 small molecule inhibitor IPA-3 combined with traditional chemotherapy drugs could significantly increase the inhibition rate and apoptosis rate of AML cells.

### PAK1 Plays a Critical Role in Stroma-Mediated Protection of AML Cells

**FIGURE 5:**
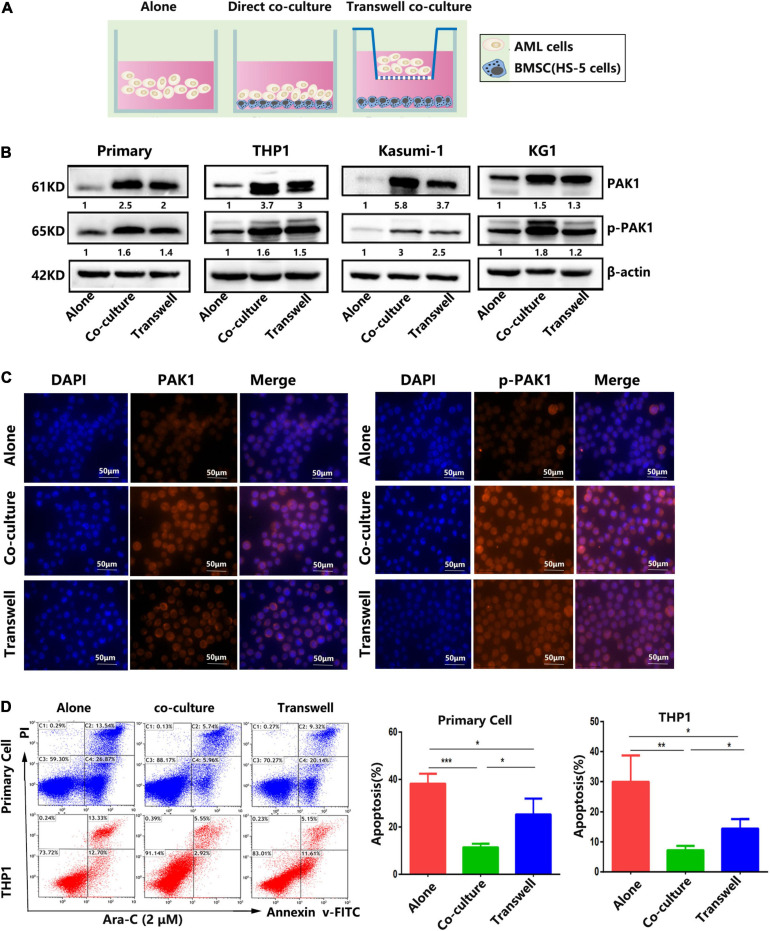
Bone marrow stromal cells confer resistance to Ara-C-induced apoptosis in AML cell lines and primary AML cells. **(A)** AML cells were cultured alone or with bone marrow stromal cells HS-5 (1:1) in a direct contact co-culture system (co-culture) or an indirect transwell co-culture system (transwell). **(B)** Primary AML cells and AML cell lines (THP1, Kasumi-1, and KG1) were cultured alone or with HS-5 for 24 h. Western blot was performed to evaluate the expression levels of PAK1 and p-PAK1 in AML cells. **(C)** THP-1 cells were cultured alone or co-cultured with HS-5 cells for 24 h, and immunofluorescence was utilized to analyze the expression of PAK1 and p-PAK1 protein. Representative images of nucleus, PAK1/p-PAK1 staining and merged images of nucleus and PAK1/p-PAK1 were shown. **(D)** Primary AML cells and THP1 cells were cultured alone or with HS-5 overnight, and then AML cells were treated with 2 μM Ara-C for 24 h. Flow cytometry was performed to analyze the apoptotic rate. All the experiments were repeated for three times. *P < 0.05, **P < 0.01, ***P < 0.001.

Accumulating evidence indicates that the interaction between hematological malignancies and BMM plays an important role in drug resistance by secreting various chemicals and contacting signals (Mendez-Ferrer et al., 2020). Among the components of the BMM, bone marrow-derived mesenchymal stem cells (BMSCs) are the most important factor, which is involved in tumor survival and drug resistance (Ridge et al., 2017). In this study, we found that the expression of PAK1 in AML cells were significantly increased when AML cells were co-cultured with BMSCs directly or indirectly. As shown in Figure 5A, primary AML cells and AML cell lines (THP1, Kasumi-1, and KG1) were cultured with BMSCs HS-5 in a direct contact co-culture system or an indirect transwell co-culture system. Western blot showed that PAK1 as well as phosphorylated PAK1 (p-PAK1) of AML cells were overexpressed in co-culture systems compared with AML cell culture alone (Figure 5B). Immunofluorescence analysis also verified that the fluorescent expression of PAK and p-PAK1 was enhanced when AML cells were co-cultured with BMSCs (Figure 5C). Moreover, the expression levels of PAK1 and p-PAK1 seemed to be even higher in the direct co-culture system than in the indirect transwell system. Meanwhile, the apoptosis of AML cells induced by Ara-C was significantly inhibited in both co-culture systems compared with AML cell culture alone (Figure 5D). And the apoptotic rate of AML cells was even lower in the direct co-culture system than in the indirect transwell system, which was oppositely consistent with PAK1 expression (Figure 5D). Our results indicates that PAK1 may exert a significant role in BMSC mediated drug resistance in AML cells.

### PAK1 Suppression Reverses the Stroma-Induced Drug Resistance in AML Cells

**FIGURE 6:**
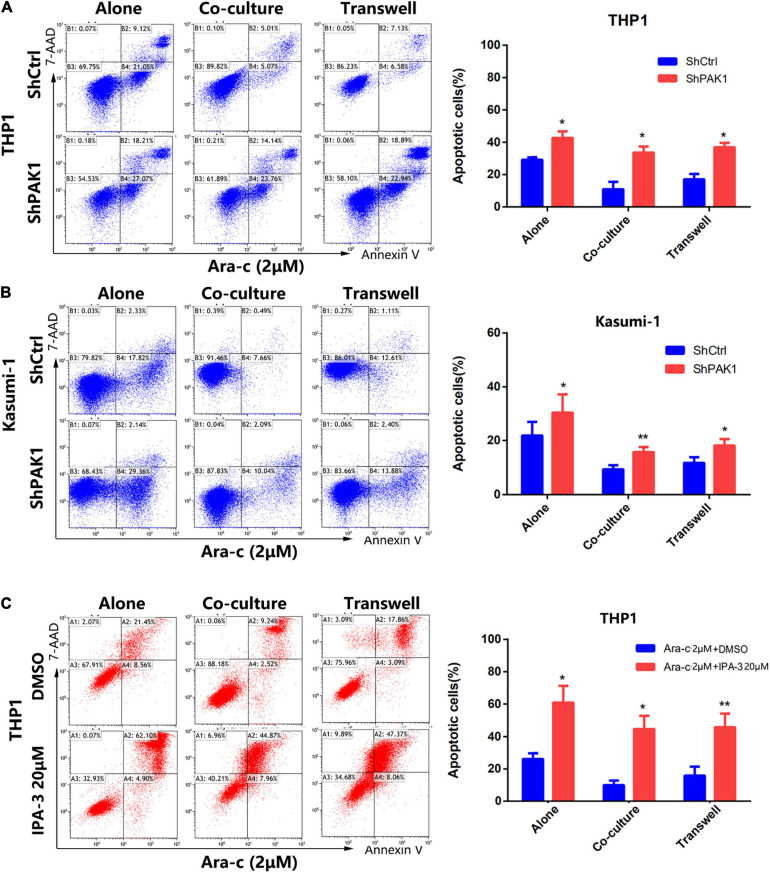
p21-activated kinase 1 (PAK1) suppression reverses the stroma-induced drug resistance in AML cells. **(A,B)** The PAK1 expression in THP1 and Kasumi-1 cells was knocked down by PAK1 shRNAs (ShPAK1). Then cells were cultured alone or co-cultured with HS-5, including direct and indirect transwell contact. Cells were treated with 2 μM Ara-C for 24 h, and the apoptotic rates of AML cells were detected by flow cytometry. **(C)** The THP1 cells were cultured alone or co-cultured with HS-5, including direct and indirect transwell contact. After exposure to IPA-3 (20 μM) and Ara-C (2 μM) for 24 h, the apoptotic rates were detected by flow cytometry. All the experiments were repeated for three times. *P < 0.05, **P < 0.01.

To further elucidate whether the stroma-induced drug resistance of AML cells is due to the activation of PAK1, THP1 and Kasumi-1 cells were transfected with PAK1 shRNAs and then co-cultured with HS-5 cells. After exposure to Ara-C (2 μM) for 24 h, cell apoptosis of co-cultured cells was examined by flow cytometry. As shown in Figures 6A,B, after knockdown of PAK1 by shRNAs, the apoptosis of co-cultured AML cells induced by Ara-C was dramatically increased. Moreover, the same results were also observed in the co-cultured THP1 cells after cells were treated with PAK small molecule inhibitor IPA-3 (20 μM) (Figure 6C). These findings suggested that PAK1 played an important role in the protection of BMSCs against AML cells. Inhibition of PAK1 could attenuate the protective effect of BMSCs on AML cells and increase the sensitivity of AML cells to chemotherapeutic agents.

### PAK1 Promotes AML Chemoresistance by Activating the ERK Signaling Pathway

**FIGURE 7:**
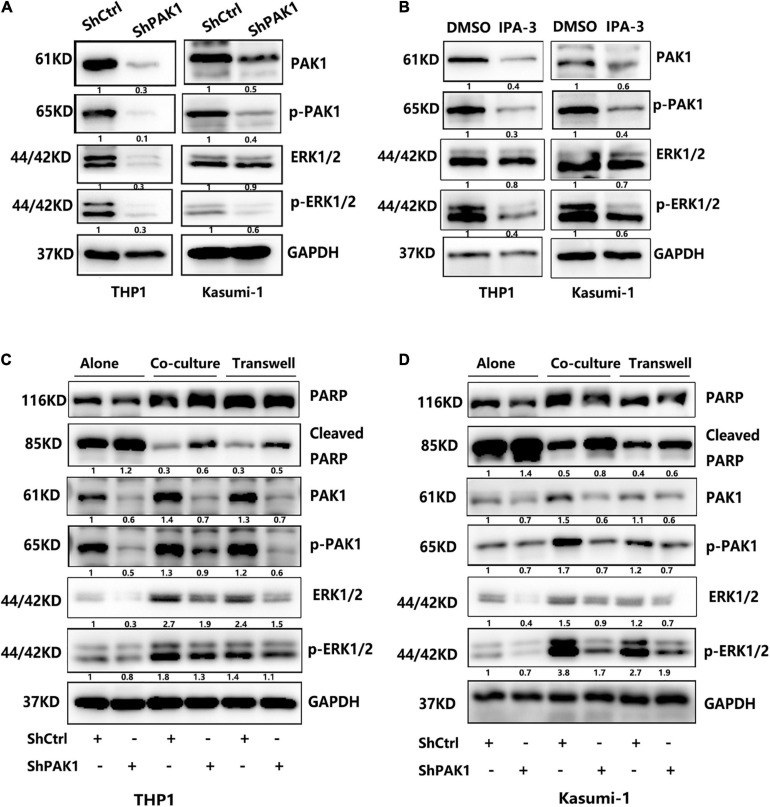
p21-activated kinase 1 (PAK1) works by activating the ERK signaling pathway. **(A)** THP1 and Kasumi-1 cells were transduced with lentivirus containing PAK1 shRNAs (ShPAK1) or scrambled control (ShCtrl). Then, the proteins of ERK1/2 and phosphorylated ERK1/2 (p-ERK1/2) were analyzed by western blot. GAPDH was used as an internal loading control. **(B)** THP1 and Kasumi-1 cells were treated with PAK inhibitor IPA-3 (20 μM) for 24 h and western blot was performed to evaluate the expression levels of ERK1/2 and p-ERK1/2. **(C,D)** THP1 and Kasumi-1 cells transduced with ShPAK1 or ShCtrl were cultured alone or co-cultured with HS-5, including direct and indirect transwell contact, and western blot was used to detect the expression of PAK1, p-PAK1, ERK1/2, p-ERK1/2, and PARP, cleaved-PARP. GAPDH was used as an internal loading control.

We subsequently tried to figure out the mechanism of PAK1 in AML chemoresistance. Previous studies showed that PAK1 was innately associated with ERK pathway in various tumors, such as melanoma, breast cancer, malignant peripheral nerve sheath tumors and Non-small cell lung cancer (Ong et al., 2013; Semenova et al., 2017; Kanumuri et al., 2020; Song et al., 2021). But there is no report on whether ERK signaling pathway is activated in AML. Accordingly, we further explored the regulation of PAK1 on ERK signaling pathway in AML cells. We used GEPIA and TCGA datasets to analyze the relationship between PAK1 and ERK1/2 mRNA expression in AML patients. The results verified that ERK1 and ERK2 expression were positively correlated with PAK1 expression in AML patients (*P* < 0.001, Supplementary Figure 2). We found that the expression of phosphorylated ERK1/2 (Thr202/Tyr204) were remarkably reduced after inhibition of PAK1 by shRNAs or IPA-3 in THP1 and Kasumi-1 cells (Figures 7A,B). When AML cells were co-cultured with BMSCs, the expression level of phosphorylated ERK1/2 was also significantly upregulated compared with cultured alone, which was the same as that of PAK1 and p-PAK1 (Figures 7C,D). Furthermore, p-ERK1/2 expression was also decreased after knockdown of PAK1 in AML cells co-cultured with BMSCs. Additionally, we also evaluated the expression of cleaved-PARP, an important apoptosis molecule. Regardless of whether it was cultured alone or in a co-culture system, the expression of cleaved-PARP was significantly increased when knocking down the PAK1 expression. Since the ERK pathway is pivotal for regulating cell proliferation, apoptosis and chemoresistance, our results implied that the roles of PAK1 in those process were partially attributed to the activation of ERK signaling pathway.

## Discussion

Acute myeloid leukemia is the most common malignant myeloid disorder in adults, and is characterized by clonal heterogeneity of hemopoietic progenitor cells. Front-line induction therapy with cytarabine and anthracycline remains a standard of care in AML (Tallman et al., 2019). With the sequential emergence of gene-targeted drugs and the development of cell therapy and biological immunotherapy, the complete remission rate and survival rate of leukemia patients have markedly improved compared with the past (Dombret and Gardin, 2016). However, there are still considerable patients with treatment failure or disease recurrence, which is closely related to the resistance of chemotherapy drugs. In recent years, the drug resistance-related mutations, especially its interaction with the tumor microenvironment, has gradually become a hot spot in tumor research. For instance, Konopleva et al. (2002) demonstrated that stromal cells prevent apoptosis of AML cells by up-regulation of anti-apoptotic proteins, including BCL2. Additionally, bone marrow stroma can mediate drug resistance to FLT3 inhibitors by persistent activation of extracellular regulated kinase in FLT-ITD AML (Yang et al., 2014).

The PAKs are a family of serine-threonine kinases that consist of six members (PAK1-PAK6), which are overexpressed in variety of cancers. PAK1 and PAK2 are overexpressed in breast and liver cancer, and promote the occurrence and development of tumors (Ong et al., 2015; Rane and Minden, 2019). PAK3 currently has been found to be overexpressed in neuroendocrine tumors (Liu et al., 2010). High expression of PAK4 promotes myeloma cell proliferation through activation of multiple myeloma anti-apoptotic and survival pathways (Fulciniti et al., 2017). By analyzing the data of newly diagnosed AML patients from the TCGA and public GEPIA database, we found that only PAK1 is significantly up-regulated in primary AML patients and confers to poor survival of AML compared with other PAK family members. Our findings were in accordance with the studies of Pandolfi et al. (2015). In addition, PAK1 gene amplification or protein overexpression was also observed in many other kinds of tumors, including breast cancer, colorectal cancer, and hepatocellular carcinoma (Kawahara et al., 2012; Xu et al., 2012; Song et al., 2015). It is well acknowledged that genetic and cytogenetics abnormality were responsible for the progression and prognosis of AML patients (Metzeler et al., 2016; Papaemmanuil et al., 2016; Bullinger et al., 2017). In the present study, our data also revealed that the high expression of PAK1 was intimately linked to higher cytogenetic risk and complex karyotype, serving as one of the risk factors in newly diagnosed AML patients. And we also found in AML patients without FLT3, IDH1, NPMc, or RAS mutation, high PAK1 expression indicated poor prognosis. Besides, complex karyotype, specific chromosomal aneuploidies (e.g., trisomy 8, −7/7q−) combined with FLT3-ITD mutation are identified as poor prognostic markers. Our study showed that FLT3 mutation positive patients with high PAK1 expression had the shortest survival time, but this was not statistically different from FLT3 mutation negative patients with high PAK1 expression. Previous studies reported that PAK1 was involved in drug resistance of multiple tumors (Yao et al., 2020). Lu et al. (2017) investigated that PAKs became activated in cells with acquired drug resistance and have a pivotal role in mediating resistance. In BRAFi-resistant cells of metastatic melanoma, PAKs phosphorylated CRAF and MEK to reactivate ERK. Gonzalez et al. (2017) reported that pharmacological inhibition of RAC1-PAK1 axis could enhance tamoxifen sensitivity in human resistant breast cancer cells. PAK1 inhibitor remarkably attenuated tumor growth and metastasis *in vivo*, and the combined treatment containing synergistic inhibition of PAK1 benefits the tumor therapy. Flis et al. (2019) confirmed that repression of PAK1 displayed synergistic effect with tyrosine kinase inhibitors in chronic myeloid leukemia. In this study, we experimentally validated that knockdown of PAK1 could reduce cell proliferation and promote AML cell apoptosis. Our studies also showed that down-regulation of PAK1 significantly increased the apoptotic effect of the chemotherapy drugs Cytarabine and Idarubicin in AML. The data revealed that the inhibition of PAK1 could enhance chemosensitivity, highlighting the crucial role of PAK1 in drug resistance of AML.

In addition to genetic abnormalities, the BMM also exerts important influences on the chemotherapy resistance of hematological tumors. Previous reports have revealed that bone marrow stroma contributes greatly to the development of drug resistance to chemotherapy in multiple myeloma, acute, and chronic myelogenous leukemia (Konopleva et al., 2002; Nefedova et al., 2003, 2004). BMSCs can protect leukemia cells against chemotherapy drugs through various cytokines or growth factors (e.g., PDGF, VEGF, EGF, SCF, etc.) or cell-cell surface contact (e.g., SDF1/CXCR4, VLA4/VCAM1, CD44/E-selectin, etc.) (Manabe et al., 1994; Wilson and Trumpp, 2006; Weisberg et al., 2008; Zeng et al., 2009; Sison and Brown, 2011). In our study, the co-culture system of BMSCs HS-5 and acute leukemia cells, including direct co-culture and indirect transwell co-culture to mimic the BMM was established, and the Annexin V-FITC analysis showed that HS-5 obviously suppressed the apoptosis of AML cells induced by Ara-c. Interestingly, the qRT-PCR and western blot results demonstrated that co-culture with BMSCs induced PAK1 expression in AML cells, and knockdown of PAK1 in AML cells partially reversed the BMSCs-induced resistance to Ara-c by apoptotic analysis. There are also some literatures reports that PAK1 may play a critical role in stroma-mediated protection of solid tumor cells. For example, Yeo et al. (2017) found that knockout of PAK1 in the stroma led to a decrease in PAK1 levels and activity, and subsequent tumor growth inhibition in pancreatic cancer. Additionally, the expression level of PAK1 in tumors was also shown to affect PAK1 levels in the stroma. Thus, PAK1 appears to have a role in signaling from the stroma to the tumor, and from the tumor to the stroma. Our data revealed that the BMSCs-mediated drug resistance may partially depend on the PAK1 activity. But how BMSCs induce the PAK1 expression and thus mediate drug resistance of AML cells requires further study.

It is well known that PI3K/AKT and MEK/ERK signaling pathways play critical roles in the interaction between the BMSCs and tumor cells (Bertrand et al., 2005; Tabe et al., 2007). Previous studies demonstrated that PAK1 could be crucial for PI3K/AKT and MEK/ERK signaling pathways, and blockage of PAK1 alleviates the tumor cells malignant behaviors via inhibiting ERK and AKT signaling activity in multiple malignant tumors (Semenova et al., 2017; Kanumuri et al., 2020; Song et al., 2021). PAK1 makes itself a contribution in this network by activating ERK, MEK, and Raf (Higuchi et al., 2008). In this study, we also found that in the BMSCs co-culture system, downregulation of PAK1 could inhibit the expression of p-ERK by western blot. Moreover, the apoptotic factor cleaved-PARP expression was also markedly increased. These data indicated that BMSCs-mediated chemotherapy-resistance may be ascribed to the PAK1 activated ERK1/2-cleavd-PARP signaling pathways.

In conclusion, in the PAK family, PAK1 overexpression confers to the shorter survival and poor prognosis of AML patients. The knockdown of PAK1 could reverse chemoresistance by induction of apoptosis in AML cells. Additionally, PAK1 plays critical roles in stroma-mediated protection of AML cells, which results in BMSCs-mediated apoptosis tolerance by activating ERK1/2 signaling pathway. Our data support the strategy of targeting PAK1 to overcome chemoresistance in AML, suggesting that the combination of inhibiting PAK1 and traditional chemotherapy drugs represents a potentially novel approach. However, due to the complexity and heterogeneity of the tumor microenvironment, as well as the crosstalk among oncogenic signaling pathways, the PAK1 mediated downstream molecular mechanism need deeply investigated.

## Data Availability Statement

Publicly available datasets were analyzed in this study. This data can be found here: The Cancer Genome Atlas (TCGA) database/Gene Expression Profiling Interactive Analysis (GEPIA).

## Ethics Statement

Informed consent was obtained in accordance with the Declaration of Helsinki. All laboratory experiments with primary samples were reviewed and approved by the Medical Ethics Committee of Qilu Hospital, Shandong University. The patients/participants provided their written informed consent to participate in this study.

## Author Contributions

MJ and BL conceived and designed the experiments and drafted the manuscript. RJ, WeL, YZ, SD, WěL, and ML performed the *in vitro* experiments. DG and QT took responsibility for statistical analyses and interpretation of data. TS and DM collected specimens and prepared the figures. CJ took responsibility for full-text evaluation and guidance, finally approval of the version to be submitted. All authors have read and approved the final manuscript.

## Conflict of Interest

The authors declare that the research was conducted in the absence of any commercial or financial relationships that could be construed as a potential conflict of interest.

## Supplementary Material

Supplementary Figure 1 **(A)** THP1 cells were infected with lentiviral particles with specific PAK1 shRNAs (ShPAK1) or scrambled control (ShCtrl) for 48 h. Transfection efficiency was evaluated by fluorescence microscope. **(B)** THP1 and Kasumi-1 cells were infected with lentiviral particles with ShPAK1 or ShCtrl for 48 h. Transfection efficiency was evaluated by flow cytometer.

Supplementary Figure 2 The expression of ERK1, ERK2, and PAK1 in AML patients were shown as scatter diagram (from GEPIA and TCGA datasets). ERK1 and ERK2 were positively correlated with PAK1 expression in AML patients.
