# Supplementary figures and images for: PAK1 Mediates Bone Marrow Stromal Cell-Induced Drug Resistance in Acute Myeloid Leukemia via ERK1/2 Signaling Pathway
Source: Front Cell Dev Biol. 2021 Jul 8;9:686695. doi: 10.3389/fcell.2021.686695 (PMC8297649; doi:10.3389/fcell.2021.686695)

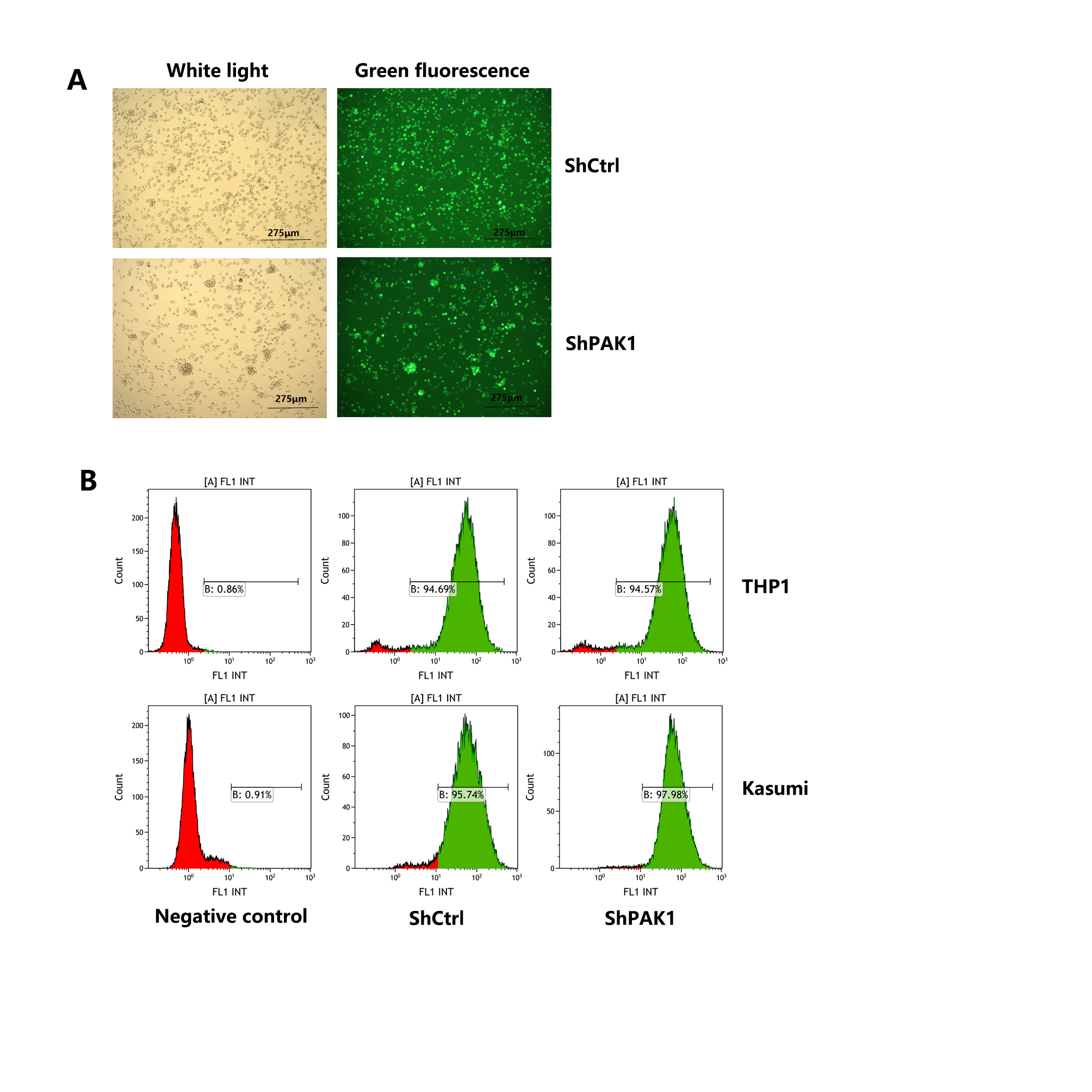

Supplement: Supplementary Figure 1 — (A) THP1 cells were infected with lentiviral particles with specific PAK1 shRNAs (ShPAK1) or scrambled control (ShCtrl) for 48 h. Transfection efficiency was evaluated by fluorescence microscope. (B) THP1 and Kasumi-1 cells were infected with lentiviral particles with ShPAK1 or ShCtrl for 48 h. Transfection efficiency was evaluated by flow cytometer. [file Image_1.TIF]

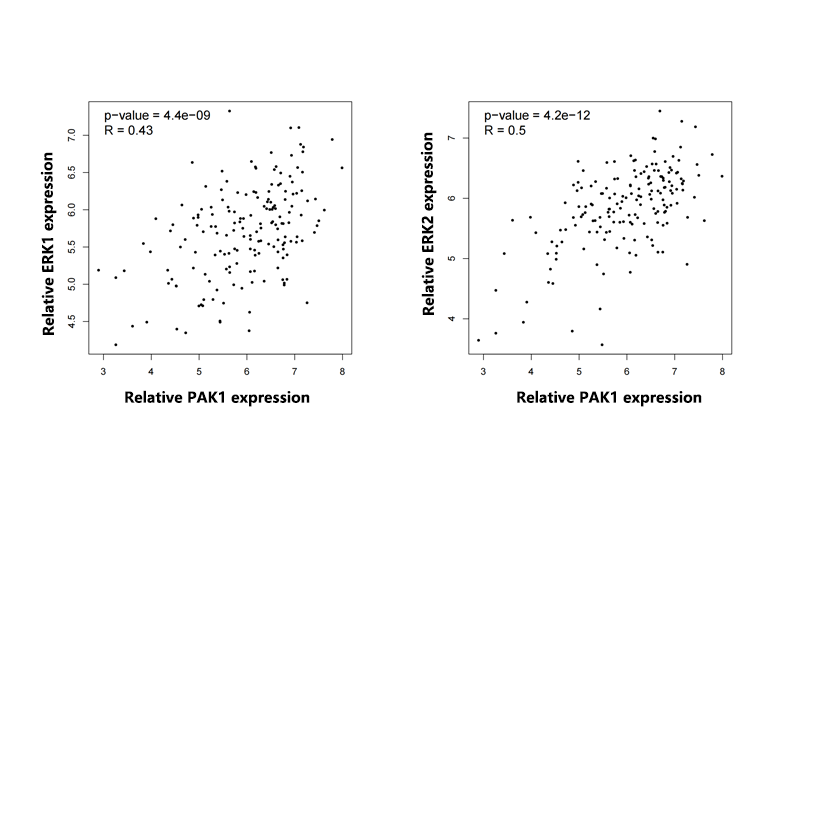

Supplement: Supplementary Figure 2 — The expression of ERK1, ERK2, and PAK1 in AML patients were shown as scatter diagram (from GEPIA and TCGA datasets). ERK1 and ERK2 were positively correlated with PAK1 expression in AML patients. [file Image_2.TIF]
